# Effects of Heat Treatment on the Interface Microstructure and Mechanical Properties of Friction-Stir-Processed AlCoCrFeNi/A356 Composites

**DOI:** 10.3390/ma16062234

**Published:** 2023-03-10

**Authors:** Shengqing Hu, Kai Wang, Simu Ma, Haoran Qi, Naijun He, Fuguo Li

**Affiliations:** 1 College of Materials Science and Engineering, Chongqing University, Chongqing 400044, China; 2 State Key Laboratory of Solidification Processing, Northwestern Polytechnical University, Xi’an 710072, China

**Keywords:** aluminum alloys, high-entropy alloys, metal matrix composites, friction stir processing, microstructure, mechanical properties

## Abstract

Equiatomic AlCoCrFeNi high-entropy alloy (HEA) has gained significant interest in recent years because of its excellent mechanical properties. A356 aluminum alloy reinforced by AlCoCrFeNi HEA particles was fabricated by friction stir processing (FSP) and subsequent heat treatment. Solution and aging treatments were specially performed for the composites to control the interface microstructure, and interfacial microstructure and tensile properties were explored at different conditions. The interface between the matrix and HEA particles showed a dual-layered core–shell structure and the thickness of the shell region increased with the solution time. The microstructure located in the shell layers consisted of a solid solution with increasing aluminum content, in which a radial-shaped solid solution phase formed in the region close to the core of the HEA particle and scattered solid solution grains with high Ni content formed in the region close to the matrix alloy. The gradient of composition and microstructure across the HEA/Al interface can be obtained through heat treatment, and an optimal interface bonding state and mechanical property were obtained after solution treatment for 2 h. Compared with FSPed A356 aluminum alloy, the FSPed composite enhanced the tensile stress by 60 MPa and the stain by 5% under the optimized conditions. The overgrowth of the shell layer decreased both the tensile strength and the ductile greatly due to the formation of a radial-shaped solid solution phase in the shell region.

## 1. Introduction

Aluminum matrix composites (AMCs) have attracted much attention for several decades due to their low density, high strength, high specific stiffness, and low cost as compared to unreinforced alloys [1,2,3]. Especially, particulate-reinforced aluminum matrix composites can satisfy various applications due to the combined properties of Al matrix and reinforcements [4,5]. Generally, ceramic particles can improve the Young’s modulus, strength, and creep resistance of aluminum, but the ductility is usually limited by their weak interface bonding [6]. Usually, a high mismatch interface between reinforcement particles and matrix leads to dislocation pile-ups and a high work hardening rate. It has been reported that the interphase between reinforcement particles and matrix can improve the interface bonding [7,8], and the coherency of the interface can solve the strength–ductility trade-off dilemma by the precipitation-assisted interface tailoring mechanism in a TiB_2_/Al-Zn-Mg-Cu composite [9].

Due to the excellent properties of high-entropy alloys (HEAs) [10,11], HEA-reinforced Al matrix composites were intensively studied recently [12,13]. Compared with ceramics, HEAs have a better interface compatibility with metal matrix and tend to form good metallurgical bonding [14]. Zhang et al. [15] reported that the good-bonding AlCoCrFeNi HEAs in 2219 Al matrix significantly improved the strength and hardness. In addition, HEA particles could also play the role of inoculant during the casting process of AMCs, refining grains and the secondary phase [16,17].

Recently, some studies showed that the balance between strength and ductility of AMCs could be solved through forming appropriate interface products during the thermal process [18,19,20]. In the (Ti-Al_3_Ti)/A356 composite, Ti particles with Al_3_Ti interface products wrapped around them formed a so-called core–shell structure, leading to an unprecedented tensile breaking elongation of 8.3 ± 0.8% [18]. The complex multi-interface of the core–shell structure can effectively weaken the stress concentration both on and inside reinforcement and restrain the propagation of the fracture [19]. The core–shell structure in HEA/Al matrix is more complicated due to the multiple elements in AMCs [21,22]. The 5% AlCoCrFeNi/Al composite with the dual-layered bilayer core–shell structure improved yield stress and plasticity by 65.1% and 42.7%, respectively, as compared with the composite without the core–shell structure [20]. At present, the specific structure of the core–shell in the AlCoCrFeNi/Al composite is still unclear. Yang et al. [23] pointed out that the shell region consisted of an Al_13_(CoCrFeNi)_4_ intermetallic and Al_9_Cr_2_ type intermetallic compound. Liu et al. [20,21,22] recognized the microstructure in the shell zone as a solid solution in AlCoCrFeNi/Al composites. The difference in the interface structure may be dependent on the properties of reinforcement and matrix alloys as well as processing methods.

Spark plasma sintering technology is a common way to prepare HEA-reinforced Al matrix composites, while a severe interface reaction might form multiple intermetallic compounds and injure the properties of HEA-reinforced AMCs due to excessive interface instantaneous current [14,23]. Comparatively, friction stir processing (FSP) is a solid-state processing technique, which can avoid the severe interface reaction [24,25]. Gao et al. fabricated an AlCoCrFeNi/5083Al composite without harmful intermetallic compounds via FSP [26], which possess higher microhardness and wear resistance as compared to the Al5083 base alloy.

Aluminum–silicon alloys are widely used in the automotive industry due to its low cost, while the strength–ductility trade-off has been a long-standing dilemma for structural application [18,19]. Composites produced by FSP can significantly improve the ductility due to microstructural refinement [27]. However, the uniform distribution of reinforcement requires a multi-pass process, resulting in a processing heat cycle and injuring the reinforcing efficiency of particles [28]. In addition, it has also been reported that a proper amount of interface products in multilayered composites can improve both strength and ductility [29,30,31]. The formation temperature of the core–shell microstructure in an HEA-reinforced Al matrix composite was reported as 560 °C [22], which is much higher than the conventional T6 heat treatment (solution + aging treatment) temperature of cast aluminum alloys. Strong milling and heat exposure during friction stir machining can effectively reduce the activation energy of materials [32]. Therefore, it is feasible to properly heat-treat the HEA/Aluminum-Silicon alloy prepared by FSP to obtain a core–shell microstructure.

In this investigation, AlCoCrFeNi HEA particles were used as reinforcement to fabricate an AlCoCrFeNi/A356 composite with A356 aluminum alloy plates by the FSP technique, and T6 heat treatment was carried out on the composites to control the interface products and aimed to improve both strength and ductility. The interfacial microstructure and mechanical properties were studied comparatively, and the evolution of the interface microstructure and the fracture behaviors of the composite were discussed.

## 2. Experimental Materials and Methods

### 2.1. Materials

The chemical composition of A356 and AlCoCrFeNi particles is presented in Table 1 and Table 2, respectively. A356 aluminum alloy plates were machined into a size of 120 mm × 70 mm × 7 mm for experiments. The AlCoCrFeNi particles were purchased from Yijin New Material Technology Co., Ltd., Beijing, China.

### 2.2. Composites Preparation

**Figure 1:**
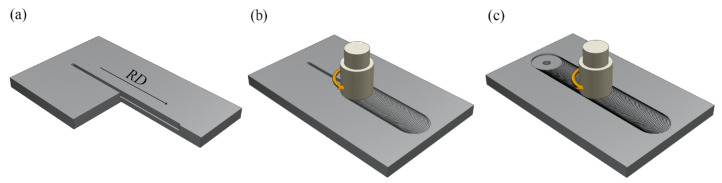

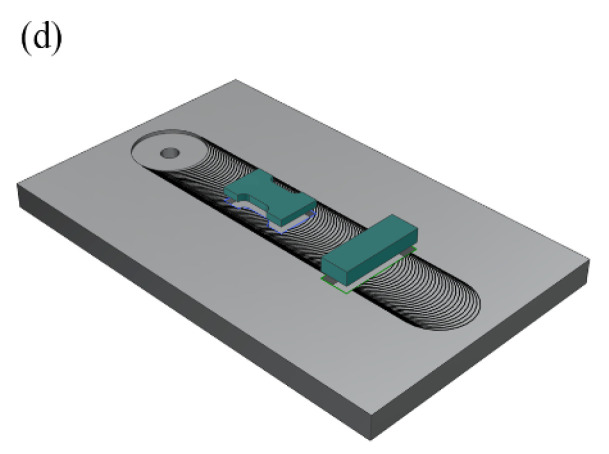
Schematic of the manufacture process of AMCs: (**a**) sectional view of pre-slotted plate, (**b**) covering the surface and particles by pinless tool, (**c**) friction stirring processing by pin tool, and (**d**) sampling locations for tensile test specimens.

**Figure 2:**
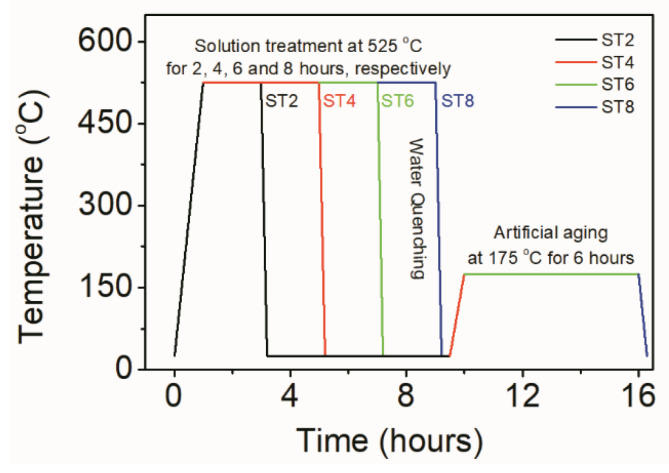
Sketch of the heat treatment processes of FSPed HEA/A356 Al composites, and FSPed A356 Al conducted solution treatment for 2 h following with the aging treatment.

Figure 1a–d show the schematic of the production processing of the AlCoCrFeNi/A356 Al composite. First, a straight groove with a length of 90 mm, a width of 2 mm, and a depth of 4 mm was machined at the middle of each workpiece plate (Figure 1a). The HEA particles were filled into the groove and pressed tightly. Secondly, a pinless rotating tool was adopted to cap the surface of the groove to avoid the spattering of reinforcement particles in the next step (Figure 1b). Finally, a three-pass FSP was carried out in the same direction along centerline of the preprocessed weld to disperse the HEA particles (Figure 1c). The pin tool for FSP was made by H13 hardened steel with a shoulder diameter of 20 mm, a pin diameter of 10 mm with a right thread, and a pen length of 4.5 mm. The parameters of rotating speed, travel speed, plunged depth, and tilting angle were 1200 rpm, 20 mm/min, 0.2 mm, and 2°, respectively. In order to adjust the interface microstructure, the FSPed AMCs were solution-heat-treated at 525 °C for different times and artificially aged at 175 °C for 6 h, and temperatures and times for the heat treatment processes were as shown in Figure 2. The composite samples were maintained at 525 °C for 2 h, 4 h, 6 h, and 8 h respectively, which were named ST2, ST4, ST6, and ST8 correspondingly. The heat treatment was conducted in a resistance furnace with an accuracy of approximately ±1 °C. For comparison, A356 alloy plates were proceeded using the same FSP parameters as well, and the FSPed Al sample underwent solution treatment for 2 h named as ST2′.

The samples with a gauge section of FSPed Al and FSPed AMCs used for tensile tests were cut from the stirred zones along the FSP advancing direction (Figure 1d). In addition, samples of the A356 aluminum plate, FSPed Al, and FSPed AMCs were cut from the cross-section perpendicular to the FSP direction according to the schematic shown in Figure 1d.

### 2.3. Characterization

Both the as-received and as-processed samples used for microstructural examination were mounted and mechanically polished. Keller’s reagent (95% distilled water + 2.5% HNO_3_ + 1.5% HCl + 1% HF) was used to reveal the microstructures of these samples. Metallographic examination was completed using optical microscopy (OM, ZEISS SUPRA 35) and scanning electron microscopy (SEM, JEOL 7800F, Tokyo, Japan) equipped with an electron back scattered diffraction (EBSD, Symmetry S3, Oxford Instruments, Oxford, UK) detector. The samples used for EBSD examination were electrically polished in a solution of 10%HClO_4_ and 90% absolute ethanol at minus 20 °C at 20 V for 25 s to produce a strain-free surface. The EBSDs were operated at an accelerating voltage of 20 keV with a scanning step size of 0.15 μm, and phase identification was performed with software Xpert Highscore 2.0 (Empyrean, PANanalytical B.V., Almelo, The Netherlands) using the reference patterns of the Joint Committee on Powder Diffraction Standards (JCPDS) 9–348 and 9–169 for α-TCP and β-TCP, respectively. The JEOL 7800 F Scanning electron microscope (SEM) equipped with an energy-dispersive spectroscopy (EDS) probe was adopted to observe the morphology of powders and the microstructure in the stirring zone (SZ), and to detect the element distribution across the HEA/Al interface. The tensile test was carried out on the universal mechanical tensile testing machine at a setting loading rate of 0.5 mm/min at room temperature. The scanning electron microscope was also used to observe the fracture morphology of tensile testing. The volume fraction of HEA particles was calculated from the optical images. During the statistical process, the influence of the Si phase and other precipitates had been eliminated through ImageJ (1.8.0). The average thicknesses of the shell in the core–shell structures were calculated according to statistical datasets of the SEM image with over 50 reinforced particles. The failure fraction of the reinforcement was calculated according to the area proportion of the core–shell structure on the fracture surface.

## 3. Results

### 3.1. Microstructural Characterization

**Figure 3:**
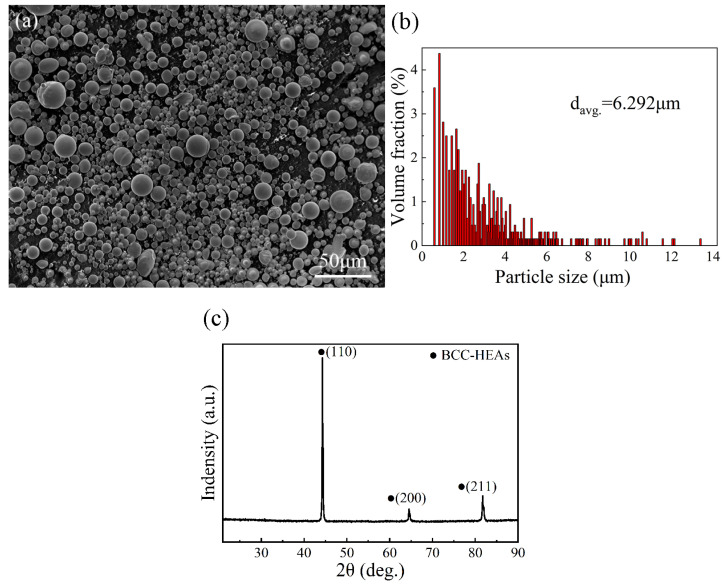
Characteristics of the as-received particles: (**a**) the SEM morphology of particles, (**b**) the size distribution, and (**c**) the XRD pattern of particles.

Figure 3 shows the features of the as-received particles, in which the as-received HEA particles were almost spherical and displayed a uniformly unimodal size distribution with an average diameter of 6.2 μm (Figure 3b). The XRD pattern of the AlCoCrFeNi HEA particles from Figure 3c presented a single BCC structure.

**Figure 4:**
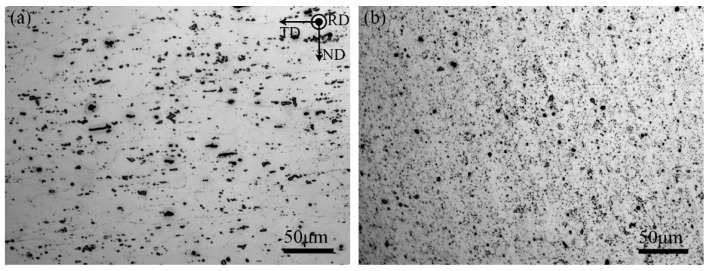
Optical micrographs showing (**a**) the microstructure of the rolled A356 aluminum plate, and (**b**) the microstructure of the stirred zone of as-FSPed A356.

Figure 4a,b display the microstructures of the rolled A356 plate and the stirred zone of the FSPed A356 plate. The rolled plate showed deformed primary grains along the transverse direction with an inhomogeneous Si particles distribution on the orientation. FSP effectively transformed the Si particles into a uniform distribution and refined the size of primary grains and Si particles.

**Figure 5:**
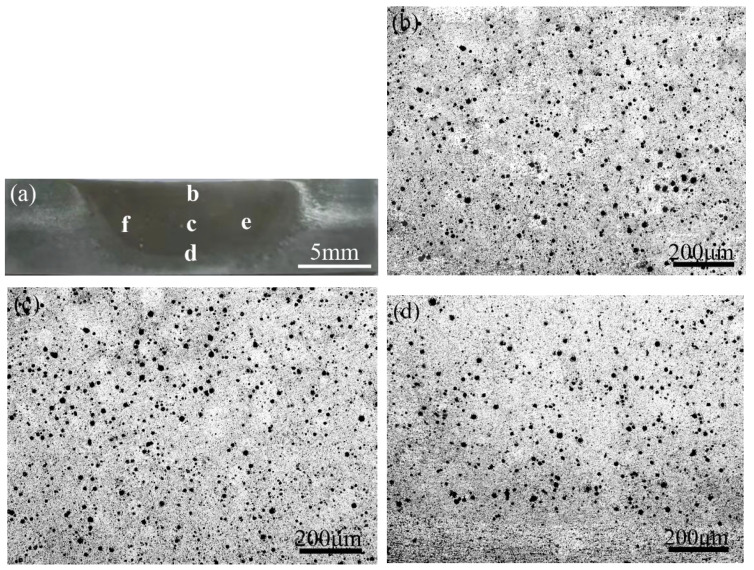

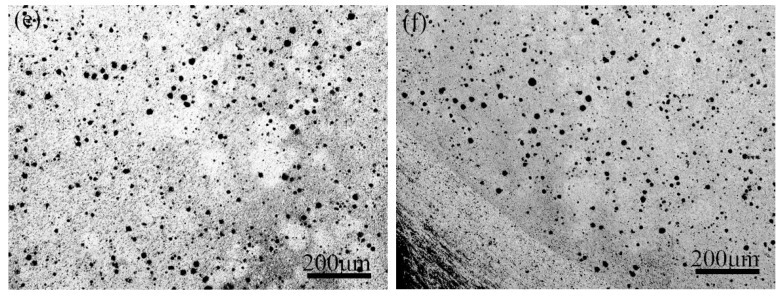
(**a**) Optical micrograph of the cross-section of the FSPed AMCs, and the microstructures in (**b**) region b, (**c**) region c, (**d**) region d, (**e**) region e, and (**f**) region f shown in Figure 5a.

Figure 5 shows the optical micrographs of FSPed AlCoCrFeNi/A356 composites on the cross-section and the microstructure from the different locations of the SZ. After a three-pass processing, the HEA particles distributed uniformly in all areas of the SZ without a marked cluster and defect. The volume fraction of HEA particles in the matrix alloy was determined as 5%.

**Figure 6:**
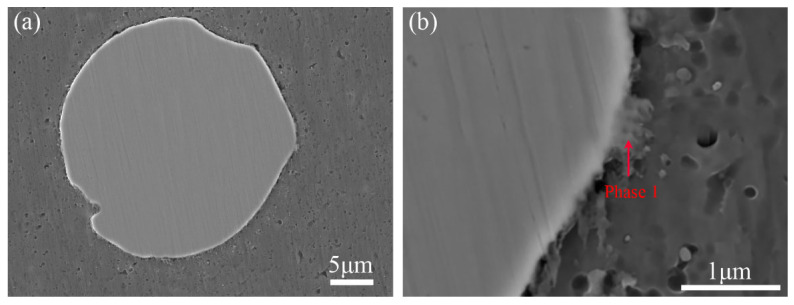

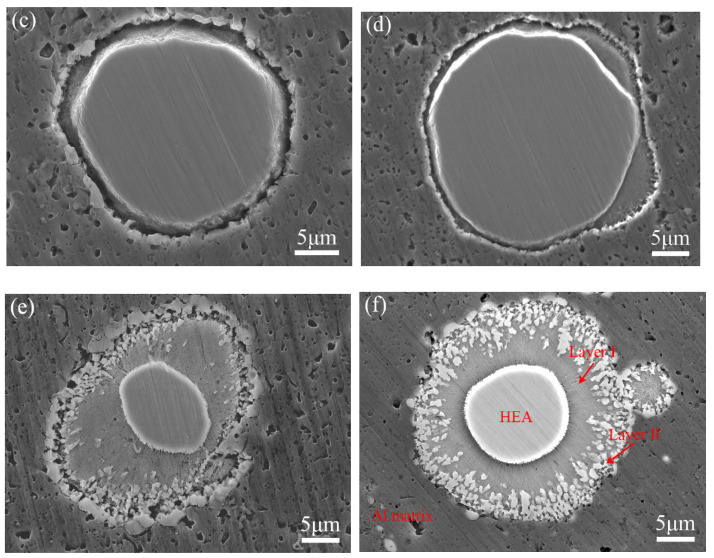
SEM images for interfacial micromorphology: (**a**,**b**) without ST, (**c**) ST2 sample, (**d**) ST4 sample, (**e**) ST6 sample, (**f**) ST8 sample.

Figure 6 presents SEM images of the interface structure between the HEA particle and Al matrix in the FSPed AMCs under different heat treatment times. The interface of the composites had a good metallurgical bonding between the HEA particle and matrix (Figure 6a), and only few gray phases marked as Phase 1 in Figure 6b could be observed at the interface, which suggested that little interface reaction between the HEA particle and the matrix metal took place during the FSP. It could be found that a ring-shaped shell zone with a two-layer structure generated around the HEA particle after solution treatment for 2 h (Figure 6c). Upon the solution treatment time increasing from 2 h to 8 h, the thicknesses of the shell zone gradually increased. Once the solution treatment time reached up to 8 h, the core part of small HEA particles ultimately disappeared due to the increase in thickness of the shell zone (Figure 6f). Generally, the microstructure of the shell zone consists of two kinds based on morphologies (Figure 6d,e), which depicts the shell zone as two layers.

Based on the microstructure shown in Figure 6f, the layer close to the core zone is named layer I, in which the morphology of the microstructure is a centripetal radial pattern; while the layer close to the matrix alloy is named Layer II, in which some white phases similar to phase 1 shown in Figure 6b scatter at the edge of this region. Owing to the generation of the shell zone during solution treatment, the sharp interface of HEA/Al in as-FSPed samples evolved into complex triple-layer interfaces at the HEA core/Layer I, Layer I/Layer II, and Layer II/Al matrix, and the dual-layered shell zone and the core zone of HEA particles exhibited a so-called core–shell structure. The average thicknesses of the shell zone in samples of SH2, SH4, SH6, and SH8 were determined as 0.9 μm, 1.4 μm, 3.5 μm, and 5.8 μm respectively.

**Figure 7:**
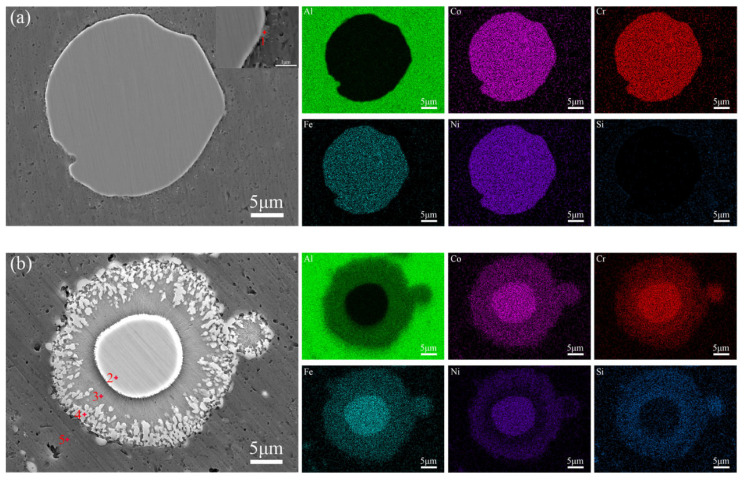
Elements distribution maps based on the interface microstructure: (**a**) the interface of as-FSPed AMCs and (**b**) the interface of ST8.

Figure 7a,b display the elemental distribution at the interface of as-FSPed AMCs. The FSPed AMCs showed distinctive elements’ content variance at the interface between the HEA core and Al matrix, although the distribution of alloy elements within the HEA particle were almost uniform. For the ST8 sample, a transition zone was formed with the decrease in content of Al and increase in content of Co, Cr, and Fe elements from the matrix to the core of the HEA particles, which corresponded to Layer I and Layer II in Figure 6f. The Ni element appeared with a little more aggregation in Layer II, while Si exhibited a markedly higher solid solubility in the shell than in the matrix. Table 3 lists the EDS results of several positions marked in Figure 7a,b to identify the constitution phases. The chemical elements of point 1 mainly consisted of Al combined with a small amount of Mg and Si, and the atomic ratios of Co, Cr, Fe, and Ni were nearly equal. The chemical elements marked as point 2 almost retained nearly atomic ratios for Co, Cr, Fe, and Ni, but the content of the Al element had a slight increase compared with that of raw HEA particles. The chemical elements located at point 3 and point 4 almost maintained approximately an equal atomic ratio of Co, Cr, and Fe with an increasing aluminum content according to Figure 7b, but the white phase marked as 4 in Figure 7b had a higher Ni content than that of point 3. Point 5 was an aluminum matrix with few Si and Mg contents. The contents of Si and Mg were almost the same for phases of point 1 and point 5, suggesting that the phase marked as 1 might be a mechanical mixture of matrix and HEA chips.

**Figure 8:**
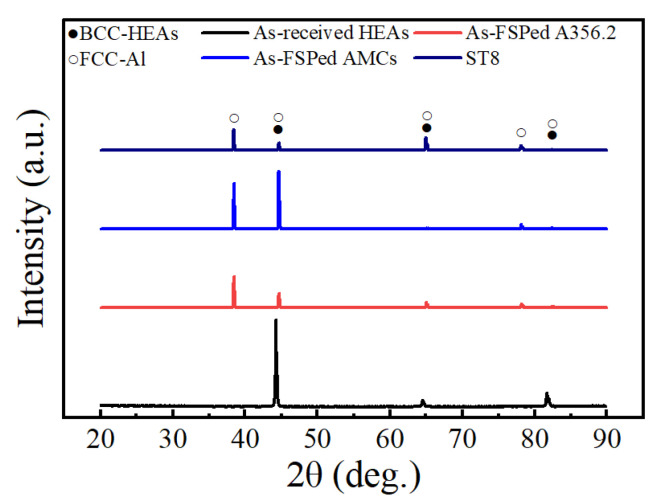
XRD patterns of samples of as-received HEA particles, as-FSPed A356, as-FSPed AMCs, and ST8.

The high-resolution XRD results of as-received particles, the as-received A356 plate, as-FSPed samples, and the ST8 sample are presented in Figure 8. Compared with the XRD results of A356 aluminum alloys, the intensity of XRD peaks of as-FSPed AMCs increased greatly at 38° and 44° for the addition of HEA particles, while they decreased to the previous level in the ST8 sample for the sake of the heat treatment. No new diffraction peaks were detected in the as-FSPed sample and the ST8 sample compared to those in the A356 alloys and HEA particles; similar results were reported by Li et al. [33]. The results suggested that HEA particles can maintain thermal stability to a certain degree, and that no new phase generated during the FSP and the heat treatment.

### 3.2. Mechanical Properties

**Figure 9:**
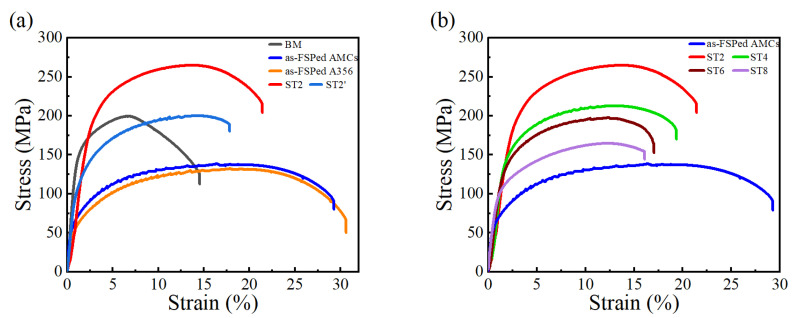
The tensile stress–strain curves of: (**a**) A356 plate, as-FSPed A356, as-FSPed AMCs, ST2 sample, and ST2′ sample; (**b**) AMCs samples with different solution time.

Figure 9 presents the tensile stress–strain curves of these studied samples, and some phenomena can be observed. First, as-FSPed A356 had a large decrease in tensile strength and a large increase in tensile strain compared with the results of the A356 plate, while the tensile strength of as-FSPed AMCs had a slight improvement over that of as-FSPed A356. Solution treatment for 2 h could improve the tensile strength greatly with a decrease in strain, and it should be noted that ST2 exhibited the largest tensile strength amongst these samples. Secondly, though the A356 plate was a deformed plate and exhibited deformed primary grains, its tensile strength and strain were much lower than those of ST2. In addition, ST2 had great increases in strength and strain by 60 MPa and 5%, respectively, compared with those of ST2′, though the strength behavior for ST2′ was the same as that of ST2 owing to the same matrix alloy and heat treatment.

Figure 9b displays the effect of solution time on the tensile stress–strain curve. ST2 had an obviously improved tensile strength with acceptable strain. When the solution treatment time increased from 2 h to 8 h, both the tensile strength and the strain of the samples decreased subsequently.

### 3.3. Fracture Morphology

**Figure 10:**
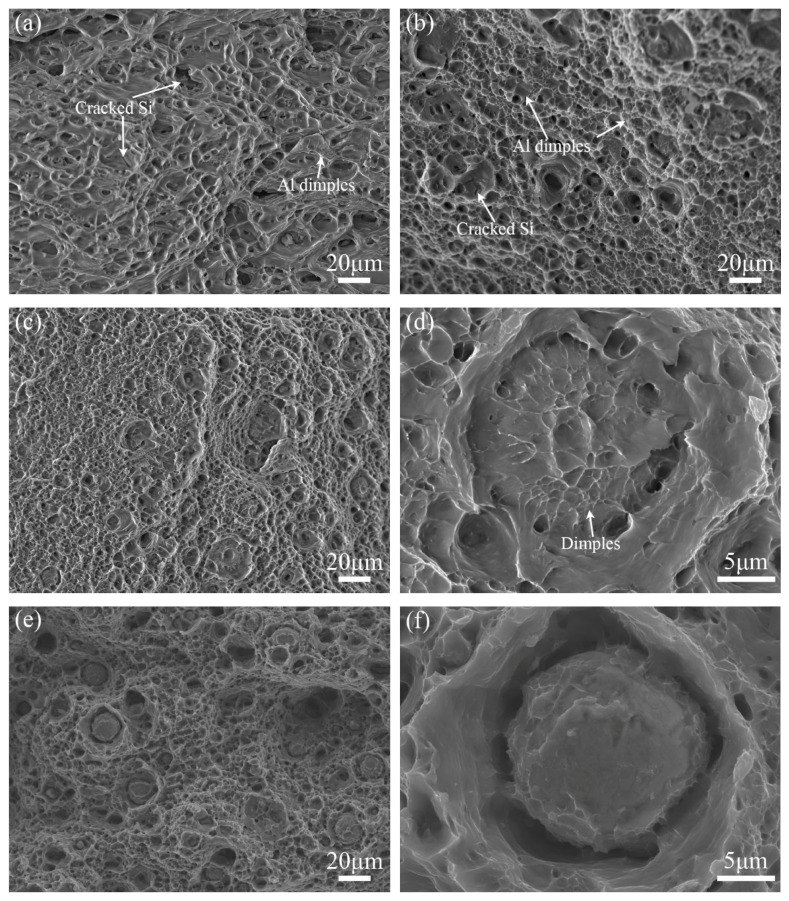

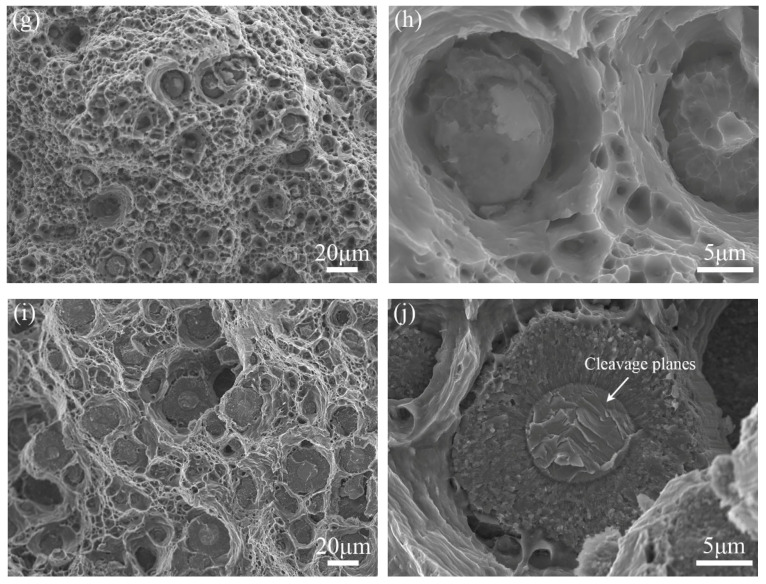
Fracture micromorphology of tensile samples: (**a**) A356 plate, (**b**) as-FSPed Al, (**c**,**d**) as-FSPed AMCs, (**e**,**f**) ST2, (**g**,**h**) ST4, and (**i**,**j**) ST8.

Figure 10 displays the tensile fracture surfaces of the A356 plate, as-FSPed Al, and as-FSPed AMCs with different solid solution times. For all these samples, many dimples could be found and had a uniform distribution on the matrix, which were of a typical ductile dimple morphology. For the fracture surface of the A356 plate shown in Figure 10a, there were also lots of cracked Si phases and holes where the Si phase was dropped on, and with bits of small dimples. In contrast with the A356 plate, the size of dimples on the fracture surface of the as-FSPed Al and AMCs appeared much finer with a more abundant number (Figure 10b,c), especially in the as-FSPed AMCs. Meanwhile, cracked Si particles still appeared on the fracture surface of the FSPed Al, but they disappeared on the fracture surface of AMCs.

For as-FSPed AMCs shown in Figure 10b, only few HEA particles could be observed on the fracture surface of the ST2. For the fracture surface of ST2 shown in Figure 10c,d, fractured particles appeared frequently on the fracture surface, and interface-induced debonding with dimples at the interface between the shell and the core of HEA particles showed on the fracture surface.

With the increase in solution treatment time, the number of fractured HEA particles increased gradually, and the fracture form of core–shell structures on the fracture surface finally evolved into brittle fracture characteristics together with many more cleavage planes on the fractured HEA particles (Figure 10j).

## 4. Discussion

### 4.1. Core–Shell Microstructure and Formation Mechanism

**Figure 11:**
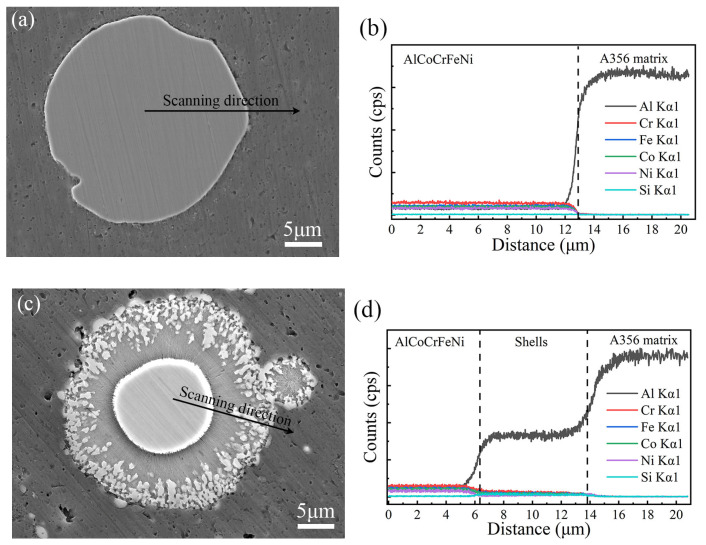
SEM-EDS line scan and the composition distribution across interface of HEA/Al (**a**,**b**) as-FSPed AMCs sample and (**c,d**) ST8 sample.

After solution treatment, ST2 exhibited a higher tensile strength and a higher strain compared with ST2′, suggesting that the evolution of the interface microstructure in the composite contributed to the improvement of both the ductility and the strength. FSP and heat treatment were applied to fabricate this AMC in this investigation, the processing temperature of the composite was low due to the solid-state processing technique for FSP [32,34,35,36], and the solution treatment was performed at 525 °C. Based on the microstructure of the as-FSPed AMCs, a core–shell microstructure was achieved for the HEA/Al interface in the composite via heating treatment, and a long solution time can lead to a wide transition zone at the edge of the HEA particles. The shell layer shown in Figure 7b had a distinct increase in aluminum content, and the contents of Co, Cr, Fe, and Ni almost maintained approximately atomic ratios (Table 3), resulting in a transition zone for the content of alloying elements from the core zone to the matrix. Figure 11 compares the distribution of alloy elements along the interface between the HEA particle and matrix, showing the results of elements linear scanning results of the as-FSPed AMCs and ST8. Both samples showed a gradient decrease for Co, Cr, and Fe and a gradient increase for Al from the core zone of HEA particles to the matrix alloy; a longer solution treatment time led to a wider transition zone.

Based on the morphological characteristics of the microstructure at the interface between HEA particles and matrix alloy, a two-layer structure was formed in the investigated AMCs, in which Layer I had only one phase and Layer II had two phases. Based on Figure 7 and Figure 11, Layer I corresponded to a Ni-poor region, while Layer II corresponded to a Ni-rich region. A reaction product was detected at the interface by TEM in the FSPed HEA/5083 composite [25], but only the HEA particle and the matrix alloys could be detected based by XRD in this research. This suggested that these phases located in the shell zone maintained the BCC-type or FCC-type lattice structure, in which the aluminum content changed greatly but the atomic ratio of Co, Cr, Fe, and Ni alloying elements almost maintained equality. The crystal structure of the AlCoCrFeNi HEA alloys depended on the aluminum content due to the lattice distortion. Hence, the change in XRD Bragg peaks intensity of the AMCs before and after solution heat treatment indicated that the phases in the shell should still be Al-based solid solution.

Considering the distribution of Ni content in the shell zone, these white grains distributed in the Layer II can be regarded as a kind of solid solution with high Ni content, and these gray phases constructing Layer I could be regarded as a kind of solid solution with low Ni content. The formation temperature of the core–shell microstructure was lower than the minimum temperature reported in previous studies, in which alloying elements in HEA began to diffuse in a large scale and to form a transition layer at interfaces between the HEA particle and the matrix alloy [20]. Obviously, the core–shell microstructure in this investigation indicated that the mechanically activated effect and heat treatment actually contributed to the formation of a shell layer.

Alloying elements’ diffusion fluxes during the heat treatment played a critical role in the formation of the core–shell structure. Based on the distribution of the aluminum element, the diffusion direction of Al, Co, Fe, Cr, and Ni was from high concentration to low concentration in the large range in the interface, so there was a gradient increase and decrease in element content from the core zone of HEA particles to the matrix alloy. The diffusive flux is always positive in the diffusion direction, and it has been known that the diffusion direction relates to the reduced chemical potential. Thus, the diffusive flux was proportional to the concentration gradient based on Fick’s first laws. Comparatively, the Al content in the matrix was about 90%, which was much greater than the Al content in the HEA particles, and the diffusion coefficient of aluminum atoms was significantly higher than that of other solute atoms in the HEA alloy [37]. However, it has been pointed out that Ni was enriched at the edge of the original HEA particle. It has been reported that the electronegativities of Cr, Co, Fe, Ni, and Al atoms are 1.66, 1.88, 1.83, 1.91, and 1.61, respectively, meaning that Ni atoms possessed a better ability to attract electrons from the surrounding Al atoms than those of other metal atoms [38,39]. The charge transfer between Ni and Al atoms was strongest, which promoted Ni to segregate at the layer I/Al matrix interface, thus reducing the total energy of the system.

The rapid diffusion of Al and the low diffusion rate of solute atoms led to faster growth of Layer I. Layer I was reported as a BCC structure [21], which was most conducive to phase stability and energy reduction for the reaction of AlCoCrFeNi/Al [33]. Additionally, the centripetal radial shape of Layer I was conducive to unidirectional heat conduction to the particles [22]. In the later stage of ST, the diffusion of the aluminum element was hindered, and the solute element diffuses outward and reacted at the Layer II/Al interface, which accelerated the formation of Layer II.

### 4.2. Strengthening and Fracture Behavior of AMCs

**Figure 12:**
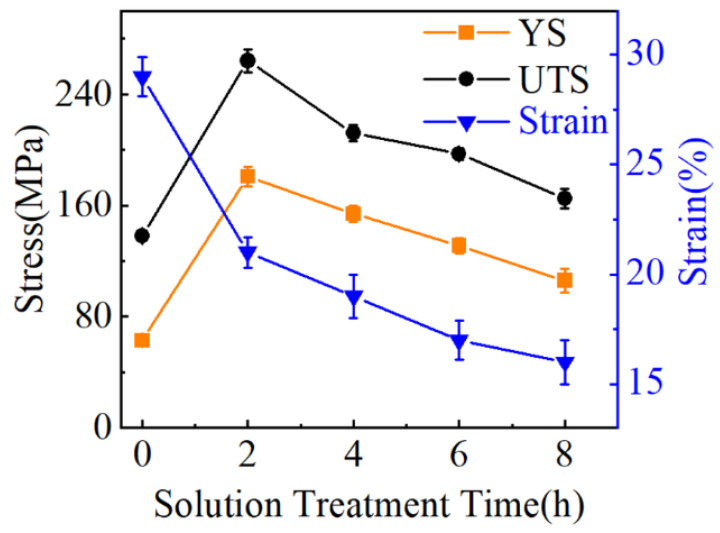
Variance of solution treatment time on tensile strength, yield strength, and stain of FSPed AMCs.

Based on the stress–strain curves shown in Figure 9, the mechanical properties of FSPed AMCs are listed in Figure 12. The tensile strength and yield strength of the composites had an obvious increase and then continuous decline with the increase in solution treatment time, but the ductility showed a progressive decline as increasing solution time. Finally, an optimized solution treatment time could be determined as 2 h, which resulted in a high tensile strength with an acceptable strain.

**Figure 13:**
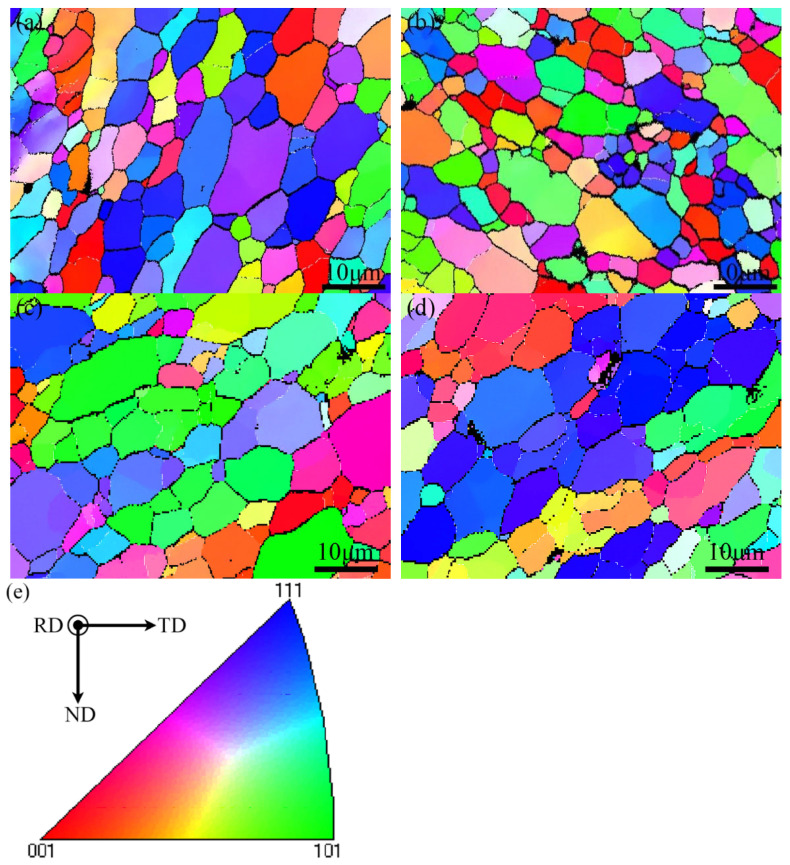
EBSD maps of: (**a**) As-Fsped Al; (**b**) As-FSPed AMCs; (**c**) ST2′ sample; (**d**) ST2 sample; (**e**) stereographic triangle of IPF color map.

In order to compare the effects of the grain size of AMCs on tensile properties, Figure 13a–d show EBSD maps of typical samples, in which high angle grain boundaries (HAGBs, misorientation angle > 15°) and low angle grain boundaries (LAGBs, 2° < misorientation angle < 15°) are drawn by bold block lines and narrow grey lines, respectively. These amounts of grains in all these samples were almost surrounded by HAGBs and maintained an equiaxed grain morphology, which suggested that they were due to dynamic recrystallization during FSP [29]. Compared to the as-FSPed A356 Al, the α-aluminum grain size of as-FSPed AMCs was much finer, which may be attributed to the pinning effects of the HEA particles on the migration of grain boundaries. In addition, these α-aluminum grains in the FSPed Al356 and the AMCs only went through a limited growth after solid solution treatment at 525 °C for 2 h. Only very small grains, which were mainly separated by LAGBs, tended to merge by adjacent large grains according to the microstructure shown in Figure 13b,d.

The mechanical properties of AMCs were generally affected by the reinforcement, the matrix alloy, and the interface microstructure. The tensile strength and elongation of the as-received A356 plate were determined as 200 MPa and 14%, respectively, and the tensile strength of the HEA alloy was reported as 396 MPa with 12% elongation [40]. The as-FSPed AMCs had an exceptional elongation, and ST2 had the strongest tensile strength together with an acceptable elongation among these studied samples. It has been proved that the core–shell microstructure occurred after solution treatment for 2 h at 525 °C. Considering the great improvement in mechanical properties of ST2 compared with ST2’, it suggested that the dual-layer interface in this AMCs resulted in the optimized mechanical properties. On the one hand, a few fine grains formed in Layer II of the shell zone, which was helpful for the increase in the total area of grain boundaries and effectively hindered the slip of dislocations, and previous research also pointed out that the hardness of the dual-layer shell zone had a higher hardness compared with Al matrix and a lower hardness than the HEA core [22]. On the other hand, the content of alloy elements in the shell zone had an optimized gradient distribution along the interface, and the solute concentration gradient established a stress gradient at the interface. Previous studies also reported that the triple interface with various grain sizes and hardness led to the redistribution of tensile stress and strain during loading, resulting in a progressive yielding of the core–shell structure [41], and long-range back stresses as well as multiaxial stress states could also generate in the heterogeneous structure of transition layers, which promoted strain hardening and delayed strain localization during plastic deformation [42]. 

However, both the tensile strength and elongation decreased continuously when the solution treatment time increased further according to Figure 10 in this investigation. It should be noted that the elongated solid solution time led to an increase in the thickness of the shell zone and, subsequently, a decrease in the core zone of the HEA particle. Principally, an enlarged shell zone should enhance the tensile strength to some degree by increasing the volume of reinforcement. However, the number and size of white grains in layer II increased greatly, and the microstructure in Layer I changed into a radial shape morphology due to the various physical properties between the HEA particles and the matrix alloy, both of which led to an adverse consequence. To sum up, the generation of the shell and the evolution of the interface microstructure can result in a great enhancement in mechanical properties for the HEA/Al composite to some degree, and an optimized interface microstructure should be designed and produced to achieve excellent mechanical properties.

**Figure 14:**
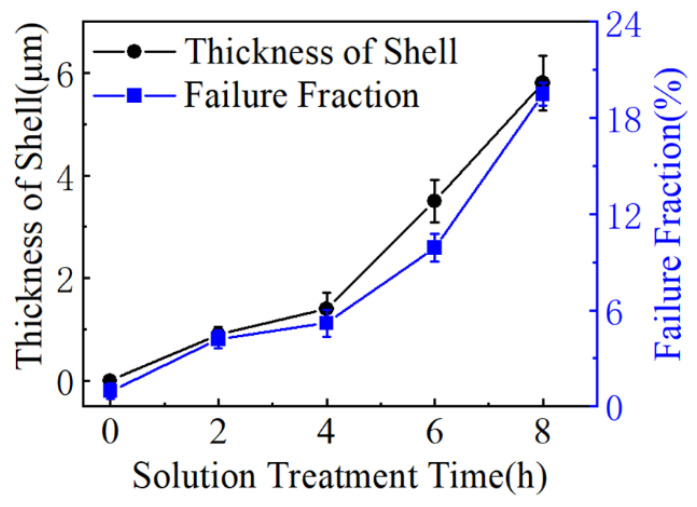
Variance of the shell thickness and failure fraction with the solution time of AMCs.

In order to examine the effects of the shell zone at the interface on the mechanical properties of samples, Figure 14 concludes the thickness of the shell zone at the HEA/Al interface in the composite and the failure fraction of HEA particles on the fracture surface under various solution treatment times. A thick shell led to a high failure fraction of fractured HEA particles. This suggests that the fracture position in the composites changed from the interface to the HEA particles, and the overgrowth of shell layers tended to decrease the effect of reinforcement and the stability of the core–shell structure.

The shell zone consists of Al-based solid solution, which has enough slip systems. Generally, the coherent boundary with low interface energy can effectively promote the movement of dislocations during deformation, while the incoherent boundary will lead to dislocation accumulation [43]. Therefore, dislocation could slip in the shell and accumulate at the incoherent Layer I/HEA core interface, leading to a stress concentration. In the early stage of deformation, the complex multi-interface microstructure contributed to the generation of back stresses and multiaxial stress states and enhanced the tensile strain [18,42]. Layer I might be a type of brittle phase due to its centripetal radial structure, which could be proved by its appearance of transgranular fracture on the fracture surface of the ST8 sample. When stress in the Layer I/HEA interface reached the critical cracking stress, a crack formed in Layer I and propagated to the HEA core and Layer II. The fine structure of Layer II could restrain the propagation of cracks via increasing the consumption of energy; therefore, the fracture of the ST2 sample showed a crack across the shell and cut through the core. The overgrowth of brittle Layer I brought about a greater stress concentration, therefore showing an increasing quantity of brittle characteristics on the core–shell stricture. However, Layer II exhibited numerous small-sized pulling-out caves, which was called the pull-out fracture mode and was favorable for energy dissipation and toughening [18,44]. Therefore, Layer I contributes an increasing restraint effect, while the overgrowth of Layer II and quantity of depleted small HEA cores result in a progressive decrease in ductility.

## 5. Conclusions

(1) The AlCoCrFeNi/A356 composite with a uniform particle distribution and limited interface reaction can be fabricated by friction stir processing. The core–shell structure between the HEA particles and the Al matrix in the composite can be controlled via atomic diffusion at high temperature.

(2) The shell zone of the interface consists of two different layers with different microstructure and element distribution, and the formation of a gradient microstructure depends on the solution diffusion and the crystal structure of HEA alloys.

(3) Microstructure and composition gradients benefit the movement of dislocation, and excellent mechanical properties can be obtained for the FSPed AMCs under an optimum solution treatment condition.

## Figures and Tables

**Table 1:** Chemical composition of the A356 aluminum alloy measured by inductively coupled plasma (ICP).

Element	Si	Mg	Fe	Mn	Ti	Al
(wt.%)	7.20	0.36	0.13	0.007	0.16	Bal.

**Table 2:** Chemical composition of the AlCoCrFeNi high-entropy alloy particles measured by ICP.

Element	Al	Cr	Fe	Co	Ni
(at.%)	22.45	19.85	19.05	20.58	18.07

**Table 3:** EDS results from different positions shown in Figure 7.

Element	Al	Co	Cr	Fe	Ni	Si	Mg
Point 1 (at.%)	91.8	1.4	1.9	1.7	1.6	0.5	1.1
Point 2 (at.%)	28.6	18.0	17.6	18.1	17.3	0.5	0.0
Point 3 (at.%)	76.9	4.4	6.1	5.4	2.1	5.0	0.0
Point 4 (at.%)	82.6	3.1	3.4	3.7	2.7	4.5	0.0
Point 5 (at.%)	98.5	0.0	0.0	0.0	0.0	0.5	1.0

## Data Availability

Not applicable.
